# Real-time fMRI data for testing OpenNFT functionality

**DOI:** 10.1016/j.dib.2017.07.049

**Published:** 2017-07-26

**Authors:** Yury Koush, John Ashburner, Evgeny Prilepin, Ronald Sladky, Peter Zeidman, Sergei Bibikov, Frank Scharnowski, Artem Nikonorov, Dimitri Van De Ville

**Affiliations:** a Department of Radiology and Medical Imaging, Yale University, New Haven, USA; b Institute of Bioengineering, Ecole Polytechnique Fédérale de Lausanne (EPFL), Campus Biotech, Geneva, Switzerland; c Department of Radiology and Medical Informatics, University of Geneva, Geneva, Switzerland; d Wellcome Trust Centre for Neuroimaging, University College London, London, UK; e Aligned Research Group, 20 S Santa Cruz Ave 300, 95030 Los Gatos, CA, USA; f Department of Psychiatry, Psychotherapy and Psychosomatics, Psychiatric Hospital, University of Zürich, Lenggstrasse 31, 8032 Zürich, Switzerland; g Neuroscience Center Zürich, University of Zürich and Swiss Federal Institute of Technology, Winterthurerstr. 190, 8057 Zürich, Switzerland; h Zürich Center for Integrative Human Physiology (ZIHP), University of Zürich, Winterthurerstr. 190, 8057 Zürich, Switzerland; i Supercomputers and Computer Science Department, Samara National Research University, Moskovskoe shosse str., 34, 443086 Samara, Russia

**Keywords:** OpenNFT, Neurofeedback, Real-time fMRI, Activity, Connectivity, Multivariate pattern analysis

## Abstract

Here, we briefly describe the real-time fMRI data that is provided for testing the functionality of the open-source Python/Matlab framework for neurofeedback, termed Open NeuroFeedback Training (*OpenNFT*, Koush et al. [1]). The data set contains real-time fMRI runs from three anonymized participants (i.e., one neurofeedback run per participant), their structural scans and pre-selected ROIs/masks/weights. The data allows for simulating the neurofeedback experiment without an MR scanner, exploring the software functionality, and measuring data processing times on the local hardware. In accordance with the descriptions in our main article, we provide data of (1) periodically displayed (intermittent) activation-based feedback; (2) intermittent effective connectivity feedback, based on dynamic causal modeling (DCM) estimations; and (3) continuous classification-based feedback based on support-vector-machine (SVM) estimations. The data is available on our public GitHub repository:

https://github.com/OpenNFT/OpenNFT_Demo/releases.

**Specifications Table**

**Table:** 

Subject area	*Neurosciences*
More specific subject area	*Neuroimaging, Real-time fMRI, Neurofeedback*
Type of data	*Data repository*
How data was acquired	*Siemens 3 T MR scanners Trio and Prisma*
Data format	*Raw, anonymized DICOMs, NIFTIs*
Experimental factors	*Approved by the local ethics committee*
Experimental features	*Real-time functional MRI*
Data source location	*Geneva, Switzerland*
Data accessibility	*The data is available under public GitHub repository:*https://github.com/OpenNFT/OpenNFT_Demo/releases

**Value of the data**

• The data allows for testing software functionality of *OpenNFT* and other neurofeedback software.
• The data allows for assessing the timing of (pre)processing steps for different feedback estimation schemes.
• The data can be used for testing the own neurofeedback setting.

## 1 Data

The three real-time fMRI data runs were acquired using (1) intermittent activation-based feedback; (2) intermittent effective connectivity feedback; and (3) continuous classification-based feedback. The interested reader can download the anonymized experimental data and re-run it using *OpenNFT* [1]. All participants gave written informed consent to participate in the experiment, which was approved by the local ethics committee. In addition to the data, we also provide files containing the *OpenNFT* settings, experimental protocol and experimental design modelled in SPM (http://www.fil.ion.ucl.ac.uk/spm).

## 2 Experimental design, materials and methods

### 2.1 Case study 1: intermittent activation-based feedback

The participant performed one fMRI localizer run to delineate bilateral primary visual cortices and a subsequent neurofeedback run to learn control over these ROIs. The localizer run consisted of eight 20 s baseline blocks that were interleaved with seven 20 s regulation blocks. During the regulation blocks, the participant was asked to perform visual-spatial imagery at a location that was indicated by a circle presented at the center of the visual field. During the baseline blocks, the participant was asked to look at the center of the screen. The neurofeedback run consisted of eight 16 s baseline blocks that were interleaved with seven 20 s regulation blocks, followed by 4 s neurofeedback display blocks. The activation-based feedback signal consisted of the scaled average activity level of the two visual ROIs. The experiment was performed at the Brain and Behavior Laboratory (University of Geneva) on a 3T MR scanner (Trio Tim, Siemens Medical Solutions, Germany). Functional images were acquired with a single-shot gradient-echo T2*-weighted EPI sequence with 150 scans (32 channel receive head coil, TR=1970 ms, volume size=74×74×36 voxels, isotropic 3 mm voxel, flip angle *α*=75°, bw=1572 Hz/pixel, TE=30 ms). The first five EPI volumes were discarded to account for T1 saturation effects.

### 2.2 Case study 2: intermittent effective connectivity feedback

The participant performed one neurofeedback run that consisted of seven trials. Each neurofeedback trial was composed of four 12 s regulation blocks interleaved with five baseline blocks of the same duration. During baseline, images of neutral objects were presented and participants were asked to passively look at them. During regulation blocks, moderately positive social images were presented and the participant was asked to experience the depicted positive social situation. The change between the conditions was indicated with the words ‘POS’ and ‘NEUT’. The feedback signal was based on a comparison of how well two alternative DCM models fitted the data acquired during a trial. The difference between the target and the alternative model dominance was computed as the difference between their logarithmic model evidence, i.e. the log of the Bayes factor (for more details, see [2], [3]). The target model represented top-down modulation from the dorsomedial prefrontal cortex (dmPFC) onto the bilateral amygdala, and the alternative model represented the bottom-up flow of information from the bilateral amygdala onto the dmPFC. The amygdala and the dmPFC ROIs were updated adaptively after each trial using an incremental GLM. The experiment was performed at the Brain and Behavior Laboratory (University of Geneva) on a 3T MR scanner (Trio Tim, Siemens Medical Solutions, Germany). Functional images were acquired with a single-shot gradient-echo T2*-weighted EPI sequence with 1050 scans (32 channel receive head coil, TR=1100 ms, volume size=120×120×18 voxels, isotropic 1.8 mm^3^ voxel size, flip angle *α*=70°, bw=1540 Hz/pixel, TE=30 ms). The first 10 EPI volumes were discarded to account for T1 saturation effects.

### 2.3 Case study 3: continuous classification-based feedback

The participant first performed two fMRI runs to provide data to train an SVM classifier using the PRONTO toolbox [4]. A single fMRI run consisted of seven 20 s regulation blocks that were interleaved with seven 20 s baseline blocks. The participant was asked to perform visual-spatial imagery during the regulation blocks, and to look at the center of the screen during the baseline blocks. Next, the participant was asked to perform a similar real-time fMRI run with a similar design (10 regulation and 11 baseline blocks), where feedback was provided as expanding circle placed at the center of the screen. The feedback signal was computed using the dot product between the pre-trained classifier weight vector and the current data vector extracted from the classification mask [5], [6]. The experiment was performed at the Campus Biotech (Geneva) on a 3 T MR scanner (Prisma, Siemens Medical Solutions, Germany). Functional images were acquired with a single-shot gradient-echo T2*-weighted EPI sequence with 210 scans (32 channel receive head coil, TR=2020 ms, volume size=100×100×35 voxels, isotropic 2.2 mm^3^ voxel size, flip angle *α*=74°, bw=1565 Hz/pixel, TE=28 ms). The first 5 EPI volumes were discarded to account for T1 saturation effects.

For more details about the applied analyses, see the associated research article [1].
