# Supplementary material for: Real-time fMRI data for testing OpenNFT functionality
Source: Data Brief. 2017 Jul 26;14:344–7. doi: 10.1016/j.dib.2017.07.049 (PMC5547236; doi:10.1016/j.dib.2017.07.049)
Supplement: Supplementary file 1 — Supplementary material [file mmc1.docx]

Conflicts of interest: none.

This is also stated in the associated Neuroimage manuscript and current DIB manuscript Acknowledgement sections.
